# Limited field radiation therapy results in decreased bone fracture toughness in a murine model

**DOI:** 10.1371/journal.pone.0204928

**Published:** 2018-10-03

**Authors:** Christopher M. Bartlow, Kenneth A. Mann, Timothy A. Damron, Megan E. Oest

**Affiliations:** Department of Orthopedic Surgery, State University of New York Upstate Medical University, Syracuse, New York, United States of America; University of Notre Dame, UNITED STATES

## Abstract

Fragility fractures are a well-known complication following oncologic radiotherapy, and it is suspected that radiation-induced embrittlement of bone within the treatment field may contribute to fracture risk. To explore this phenomenon, a mouse model (BALB/cJ) of fractionated, limited field, bilateral hindlimb irradiation (4x5 Gy) was used. The effects of radiation on femoral (cortical) bone fracture toughness, morphology, and biochemistry—including advanced glycation end products (AGEs)—were quantified and compared to Sham group samples prior to irradiation and at 0, 4, 8, and 12 weeks post-irradiation. Additionally, alterations to bone fracture toughness mediated directly by radiation (independent of cellular mechanisms) were determined using devitalized mouse cadaver femurs. Finally, the contribution of AGEs to reduced fracture toughness was examined by artificially ribosylating mouse femurs *ex vivo*. These data demonstrate that *in vivo* irradiation results in an immediate (-42% at 0 weeks, p < 0.001) and sustained (-28% at 12 weeks, p < 0.001) decrease in fracture toughness with small changes in morphology (-5% in cortical area at 12 weeks), and minimal changes in bone composition (tissue mineral density, mineral:matrix ratio, and AGE content). Irradiation of devitalized femurs also reduced fracture toughness (-29%, p < 0.001), but to a lesser extent than was seen *in vivo*. While artificial ribosylation decreased fracture toughness with time, the extent of glycation needed to induce this effect exceeded the AGE accumulation that occurred *in vivo*. Overall, hindlimb irradiation induced a substantial and sustained decrease in bone fracture toughness. Approximately half of this decrease in fracture toughness is due to direct radiation damage, independent of cellular remodeling. Collagen glycation *in vivo* was not substantially altered, suggesting other matrix changes may contribute to post-radiotherapy bone embrittlement.

## Introduction

Fragility fractures are a well-known complication following oncologic radiotherapy, occurring in up to one third of cancer survivors in certain groups [1–8]. These delayed-onset fractures can occur in bone that was captured in the radiation field months to years after radiotherapy, and are associated with impaired healing and high morbidity. While a role for radiation-associated osteopenia has been suggested, clinical studies to date have not demonstrated a conclusive connection between irradiation and decreased bone mass [9, 10]. The lack of a clear relationship between bone mineral density—measured by DXA or quantitative CT—and fracture risk suggests that changes in bone quality, including material strength, may contribute to fragility fractures. To date, however, post-radiotherapy bone embrittlement has not been quantified, and potential mechanisms regulating this embrittlement have been not been explored.

Numerous animal models have been used to study the effects of irradiation (total body, limited field, and focal) on changes to bone architecture and tissue density [11–22]. Human pathologic responses to radiation therapy (RTx), including osteocyte death, trabecular bone resorption, marrow adiposity, and increased bone fragility, are all replicated in mouse models of fractionated limited field RTx [12, 14, 21–23]. A pattern of metaphyseal trabecular bone resorption and diaphyseal cortex thinning has been observed as well [11, 21, 22]. Irradiation induced an early and persistent (4–26 weeks) decrease in mouse femur bending strength and stiffness [11]. Furthermore, material strength of cortical bone (flexural strength) was also diminished following RTx [11]. These findings suggest that changes to both structural geometry (femur diaphyseal size) and tissue properties (cortical bone strength and resistance to fracture) contribute to the overall loss of femur bending strength post-RTx. Studies to date have not, however, directly measured fracture resistance or embrittlement of irradiated cortical bone. A fracture mechanics approach—measuring crack propagation during loading [24, 25]—would provide a quantitative measure of the bone material fracture toughness (the ability of bone to resist crack propagation).

Previous work has demonstrated immediate post-RTx changes to bone collagen, including increased advanced glycation end product (AGE) content, trivalent to divalent crosslink ratios, and persistently increased matrix alignment [26, 27]. Alterations to type I collagen structure or chemistry can influence the mechanical properties of bone, including fracture toughness [28–30]. Radiation can also impact bone health through alterations in cell populations, microenvironments, and cellular function (i.e., bone formation and resorption) [22, 23, 31–34]. The relative contributions of RTx to immediate/direct (non-cell-mediated) and longer-term cell-mediated effects on biochemical and fracture toughness changes in bone are not known.

The overall goal of this study was to quantify post-irradiation changes in bone fracture toughness, morphology, and collagen biochemistry using a mouse model of fractionated, limited field hindlimb irradiation (RTx). To explore the extent to which direct radiation damage (not mediated by cells) alters biomechanics, fracture toughness was also determined in irradiated, devitalized mouse femurs. A sub-objective of this study was to determine if advanced glycation end products contribute substantively to radiation-associated changes in fracture toughness. We hypothesized that: 1) RTx induces an early and progressive reduction in cortical bone fracture toughness compared to non-irradiated (Sham) femurs; and 2) RTx induces a loss of fracture toughness directly, through non-cell-mediated mechanisms. Finally, we explored the potential for increased AGE content to reduce bone fracture toughness *ex vivo*, with comparison to the changes in AGE content following RTx *in vivo*.

## Materials and methods

### Animal model

All procedures were approved in advance by the SUNY Upstate Institutional Animal Care and Use Committee (protocol #362) in accordance with the recommendations of the *Guide for the Care and Use of Laboratory Animals of the National Institutes of Health*. Female BALB/cJ mice were procured from Jackson Labs (Bar Harbor, ME, USA) and maintained in community housing (≤ 5 mice/cage, 22°C) on a 12 h light/dark cycle with water and pellet chow (Formulab Diet 5008, LabDiet, St. Louis, MO, USA) available ad libitum. The AAALAC-accredited housing facility (Public Health Service assurance #A3514-01) provided daily welfare observations with cage and bedding changes every two weeks. A total of 172 mice were utilized in these studies, with all treatments occurring at 12 weeks of age: 137 for the *in vivo* irradiation experiment, 15 for the devitalized bone RTx experiment, and 20 for the *ex vivo* ribosylation experiment. For all studies euthanasia was completed using CO_2_ asphyxiation followed by cervical dislocation.

### *In vivo* irradiation experiment

**Fig 1:**
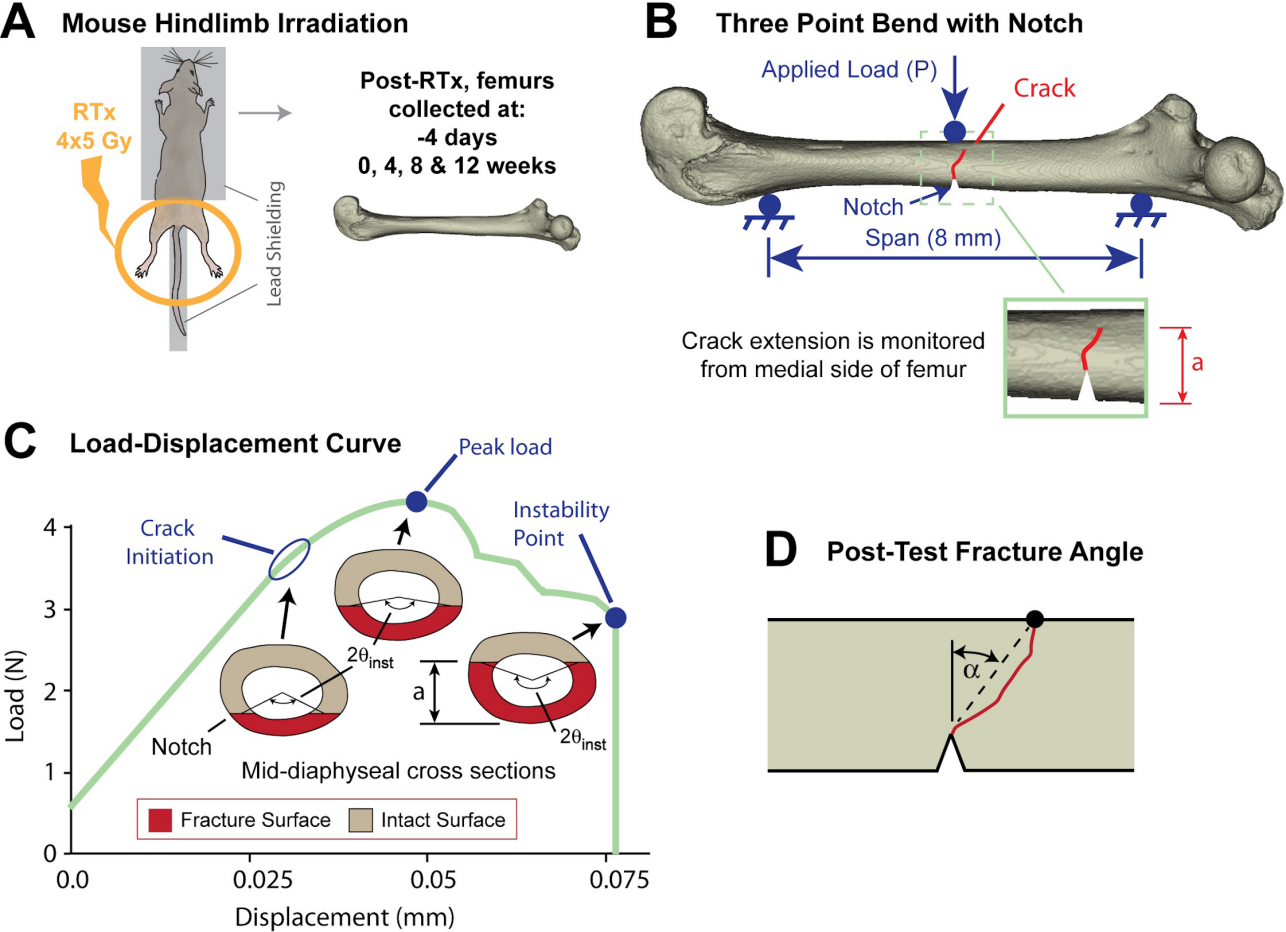
Overview of experimental design and outcome measures. (A) Animal positioning with lead shielding during irradiation and femur collection at five end points, n = 15 mice/group/end point. (B) Three-point bending configuration showing the notch created on the anterior surface of the femur; crack extension (a) is monitored from the medial side of the femur. (C) Idealized load-displacement plot for a notched femur in three-point bending showing the crack initiation, peak load, and instability points. Inset cartoons show propagation of the fracture surface (crack) corresponding to each of the toughness parameters. The instantaneous half crack angle θ_inst_ was determined at each of the three loading points to determine initiation (K_init_), peak load (K_pl_), instability (K_inst_) fracture toughness. (D) Fracture angle (α) was used as a descriptor femoral fracture pattern.

Fifteen mice were euthanized four days prior to irradiation, representing the study baseline. The remaining 122 mice were randomly assigned to RTx (n = 61) or Sham treatment groups (n = 61). The RTx group received bilateral hindlimb irradiation delivered as four consecutive daily doses of 5 Gy each (4x5 Gy). Mice were anesthetized using ketamine/xylazine (100/10 mg/kg i.p., #501090/51004, MWI Veterinary Supply, Boise, ID, USA) and positioned under 4 mm thick lead shields with hindlimbs extended into the radiation field (Fig 1A). Radiation was delivered from a collimated X-ray source (1.1 Gy/minute, 225 kV, 17 mA, 55 cm source-to-shelf distance, 0.5 mm Cu beam filter, MultiRad 225, Faxitron Bioptics, Tucson, AZ, USA). Our lab previously calculated the biologically equivalent dose for this treatment protocol to be 55.7 Gy, with the lead shielding permitting a body exposure of <0.11 Gy per 5 Gy hindlimb treatment [11]. The Sham group was subjected to the same anesthesia, handling, and recovery procedures without radiation exposure. Two mice (one Sham, one RTx) died due to anesthesia complications. Mice were euthanized at five end points: 0, 4, 8, and 12 weeks post-RTx (n = 15/group/time point). Femurs were harvested intact, stripped of soft tissues, and stored at -80°C wrapped in gauze saturated with saline.

### Irradiation of devitalized tissue

For the devitalized tissue study, 15 mice were euthanized at twelve weeks of age. Intact carcasses were frozen at -80°C for 24 hours to devitalize the tissues. After thawing the carcass, left (control) femurs were removed, cleaned of soft tissues, and stored at -80°C in saline-saturated gauze. The remaining devitalized carcasses were then subjected to unilateral right hindlimb irradiation using a single 20 Gy exposure (225 kV, 17 mA, 55 cm source-to-shelf distance, 0.5 mm Cu beam filter). Fractionated dosing was not required for devitalized tissues, as there were no viable cells requiring DNA damage repair periods. Following irradiation, the right femurs were harvested and frozen for later analysis as described above.

### Artificial ribosylation of femurs *ex vivo*

To study the effect of artificial glycation on fracture toughness, a final group of 20 mice was euthanized. Disarticulated intact femurs (n = 40) were stripped of soft tissues and randomly assigned to one of four groups: 0, 3, 7, or 14 days ribosylation (n = 10/group). Femurs were incubated at 37°C in a ribose solution (0.6 M D-ribose, 0.5 M CaCl_2_, 0.1% sodium azide, 30 mM HEPES; Sigma-Aldrich #R9629, C5670, S2002, and Corning/CellGro #61-034-RM respectively) for the pre-determined period 3, 7, or 14 days. The control group (0 days) was incubated at 37°C in a ribose-free control solution (30 mM HEPES, 0.5 M CaCl_2_, 0.1% sodium azide) for 14 days. After 3 and 7 days of ribosylation, femurs from those treatment groups were rinsed in deionized water for 1 hour and placed in control solution at 37°C until day 14. All femurs were rinsed in running deionized water for 1 hour and then stored in saline-saturated gauze at -80°C.

### Fracture toughness testing

**Fig 2:**
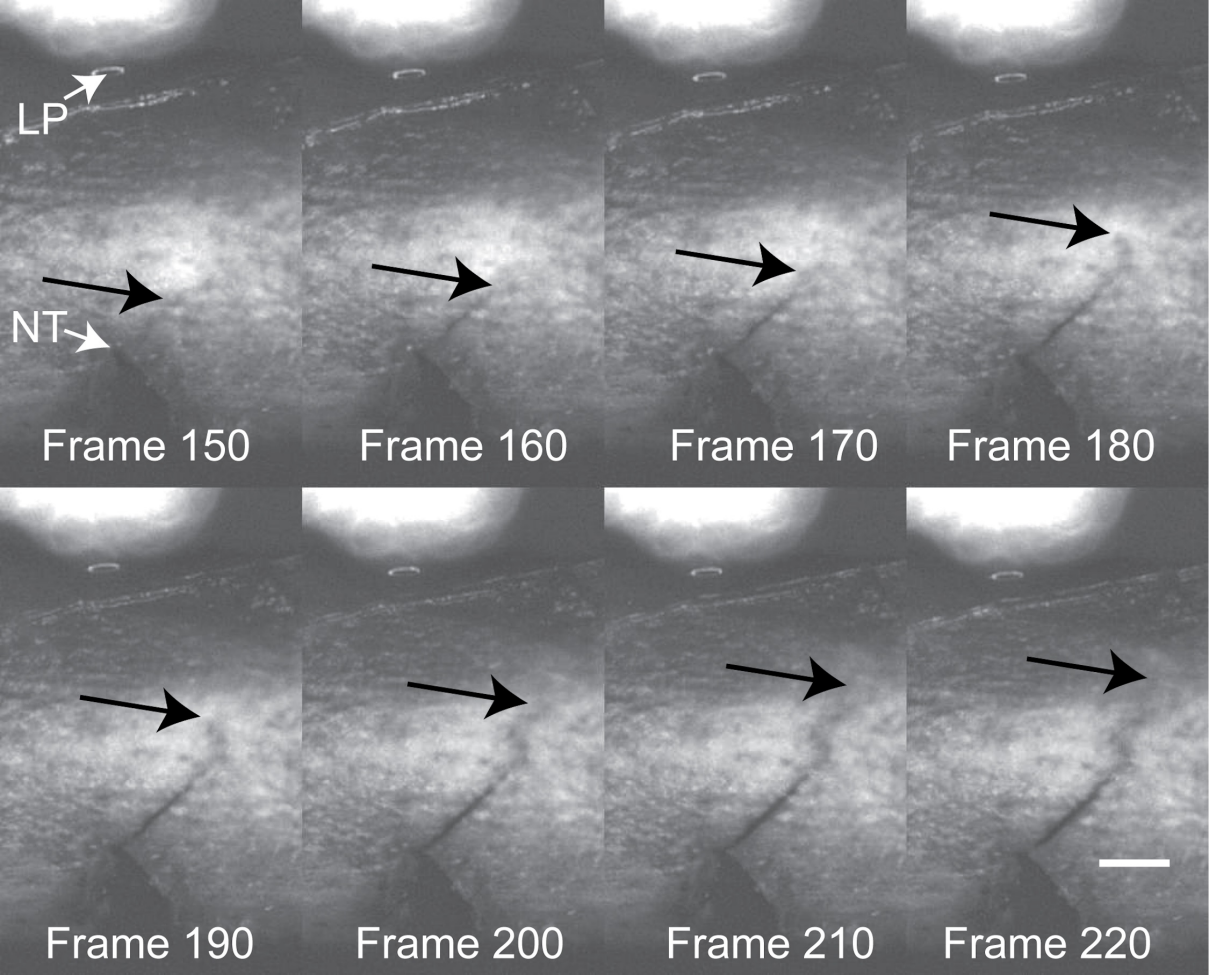
Imaging of crack tip propagation. Crack tip position (black arrows) was documented using reflected white light imaging from the notch tip (NT). The central load pin (LP) was positioned directly above the notch tip. Scale bar represents 0.2 mm.

Cortical bone fracture toughness was determined in femurs retrieved from irradiated mice (RTx), non-irradiated mice (Sham), devitalized mouse cadavers (devitalized RTx femurs and contralateral non-irradiated controls), and artificially ribosylated femurs (described above). Specimen preparation included creation of an initial notch on the anterior surface of the mid-diaphysis using a saw blade (~0.3 mm deep) followed by a platinum-coated double-edged stainless steel razor blade (~0.35 mm deep) (#72003, Electron Microscopy Sciences, Hatfield, PA) to create a sharp notch (root radii < 5 μm). Blade depths were set using custom fences that were made for each blade type. During notching, one femur from the week 4 RTx group was broken, and excluded from mechanical testing. Femurs were rehydrated in saline-filled tubes for at least thirty minutes before being placed in a three-point bending fixture with alignment verified using an optical imaging system (camera: SPOT Insight 2Mp, Diagnostic Instruments, Sterling Heights, MI, USA; lens Edmund Optics 55–910 MMS R-6 and Edmund Optics MMS OBJ-7 55–901, Edmund Optics, Gloucester, NJ, USA) with a 2.2 μm/pixel resolution, 2.64 mm x 3.52 mm field of view. This was done to ensure that the notch was centered between the two supports (8 mm span), directly below the applied load, with the notch orthogonal to the loading axis. Load was applied at the mid-diaphysis to failure, in a posterior-to-anterior direction at a rate of 0.1 mm/min at room temperature using a mechanical test frame (Qtest, MTS Corporation, Eden Prairie, MN, USA) (Fig 1B and 1C). Hydration was maintained using saline spray prior to loading; total time to failure was ≤60 seconds. Crack tip propagation from the sharp notch (Fig 2) was documented during loading (5 Hz) and image sets were synchronized with test frame load-displacement data.

The method to calculate fracture toughness during crack initiation and growth was adapted from an approach developed by Carriero et al. [25] where a crack path during mechanical loading of mouse femora was documented using environmental SEM. In this study, a high resolution imaging method was used to document the progression of crack growth, similar to the work of Katsamenis et al. [35]. The projected crack extension was measured on the medial surface of the bone (Δa_proj_), and this was used to calculate the instantaneous half crack angle (θ_inst_), which in turn, along with the instantaneous load P, and cross sectional geometry of the bone, was used to calculate fracture toughness (K). Using this data, the fracture initiation toughness (K_init_), peak load fracture toughness (K_pl_), and instability fracture toughness (K_inst_) were used to describe the crack propagation process (Fig 1C). Full details of the calculations are given in S1 Document. The nature of the diaphyseal fracture pattern was quantified using sagittal plane images of the fractured femurs. The global fracture angle (α, Fig 1D) was calculated from the root of the pre-notch to the point of fracture on the periosteal bone surface.

### Bone morphology

To document diaphyseal morphology in the vicinity of the notch, the distal half of the femoral diaphysis was imaged using micro-computed tomography (μCT) at a 12 μm isotropic voxel resolution (55 kV, 145 mA, 200 ms integration time, μCT 40, Scanco, Brüttisellen, Switzerland). An HA phantom was used as provided by the manufacturer as part of the quality control process. The mid-diaphysis was analyzed using a lower global threshold of 654 mg hydroxyapatite/cm^3^, and volumetric tissue mineral density (TMD) was quantified. Using the BoneJ plugin [36] for ImageJ [37] (NIH, Bethesda, MD, USA) mid-diaphyseal geometric parameters were measured including: cortical area (Dp Ct.Ar), mean cortical thickness (Dp Ct.Th), endosteal area (Dp Es.Ar), total area (Dp Tt.Ar), minimum moment of inertia (*I*_*min*_) and maximum moment of inertia (*I*_*max*_). The mid-diaphyseal geometry (mean diameter and wall thickness) and notch depth were determined post fracture using high-resolution white light imaging of the fracture surface. Measurements were made using ImageJ with appropriate scale calibration.

### Advanced glycation end products

After μCT imaging, distal femoral diaphyseal bone samples were hydrolyzed in 6 M HCl (48 hours at 110°C). Pentosidine and non-specific advanced glycation end products (nsAGEs) were assayed using autofluorescence (Tecan Infinite M200 plate reader, Morrisville, NC, USA). For pentosidine measurements, all samples were run in triplicate and quantified by comparison to a quinine sulfate standard curve as previously described (λ_ex_/λ_em_ 335/385 nm) [26, 38]. Measurement of nsAGEs was done without technical replicates on the same plate allowing for the relative comparison of fluorescence values (370/440 nm, 96 samples/plate, n = 10-11/group/time point). Results for nsAGEs are expressed as arbitrary fluorescence units (FU, as reported by the plate reader with gain = 100). Pentosidine and nsAGE values were normalized to the collagen content of the sample volume. Collagen content was determined in triplicate with a colorimetric hydroxyproline assay, assuming 13.5% hydroxyproline by mass [39, 40]. Mineral-to-matrix ratio was calculated using μCT-derived bone mineral content and hydroxyproline assay-derived collagen content values.

### Statistics

Descriptive statistics including mean and standard deviation were determined for each outcome measure at each time point. To test the first hypothesis, the effect of treatment (RTx vs. Sham) on cortical bone fracture toughness, composition, and diaphyseal morphology was assessed using unpaired Student’s t-tests at each time point. Analysis of covariance (ANCOVA) was used to test whether there was a progressive reduction in fracture toughness of RTx femurs (vs. Shams) with time (weeks) as a covariate. To illustrate the magnitude of the treatment effect, the percent change for outcome measures were calculated as [(RTx-Sham)/Sham] x 100.

To test the second hypothesis using devitalized mouse femurs, paired Student’s t-tests were used to test the effect of the treatment (RTx femur vs. contralateral control) on fracture toughness. One-way analysis of variance (ANOVA) with Tukey’s post-hoc tests was used to compare the fracture toughness of the devitalized femurs (RTx and control) to the week 0 groups (RTx and Sham) from the *in vivo* study. For the third hypothesis, linear regression was used to assess the effects of ribose incubation time on fracture toughness and composition. Simple linear regression was also used to assess relationships between bone composition parameters including post-RTx AGE content and fracture toughness measures. Effect size was calculated for all outcome measures using the Cohen’s d formula. All statistical analyses were run using JMP 13 software (SAS, Cary, NC, USA).

## Results

### Body mass

There were no significant differences in terminal body mass between RTx and Sham mice at the 0 or 12 week end points. At four weeks, the RTx mice weighed an average of 5% more than Sham mice (p < 0.016), while at 8 weeks the Sham mice weighed 7% more than RTx mice (p < 0.012) (S1 Table).

### Hypothesis 1: Fracture toughness is lost early post-RTx

#### Fracture mechanics outcomes

**Fig 3:**
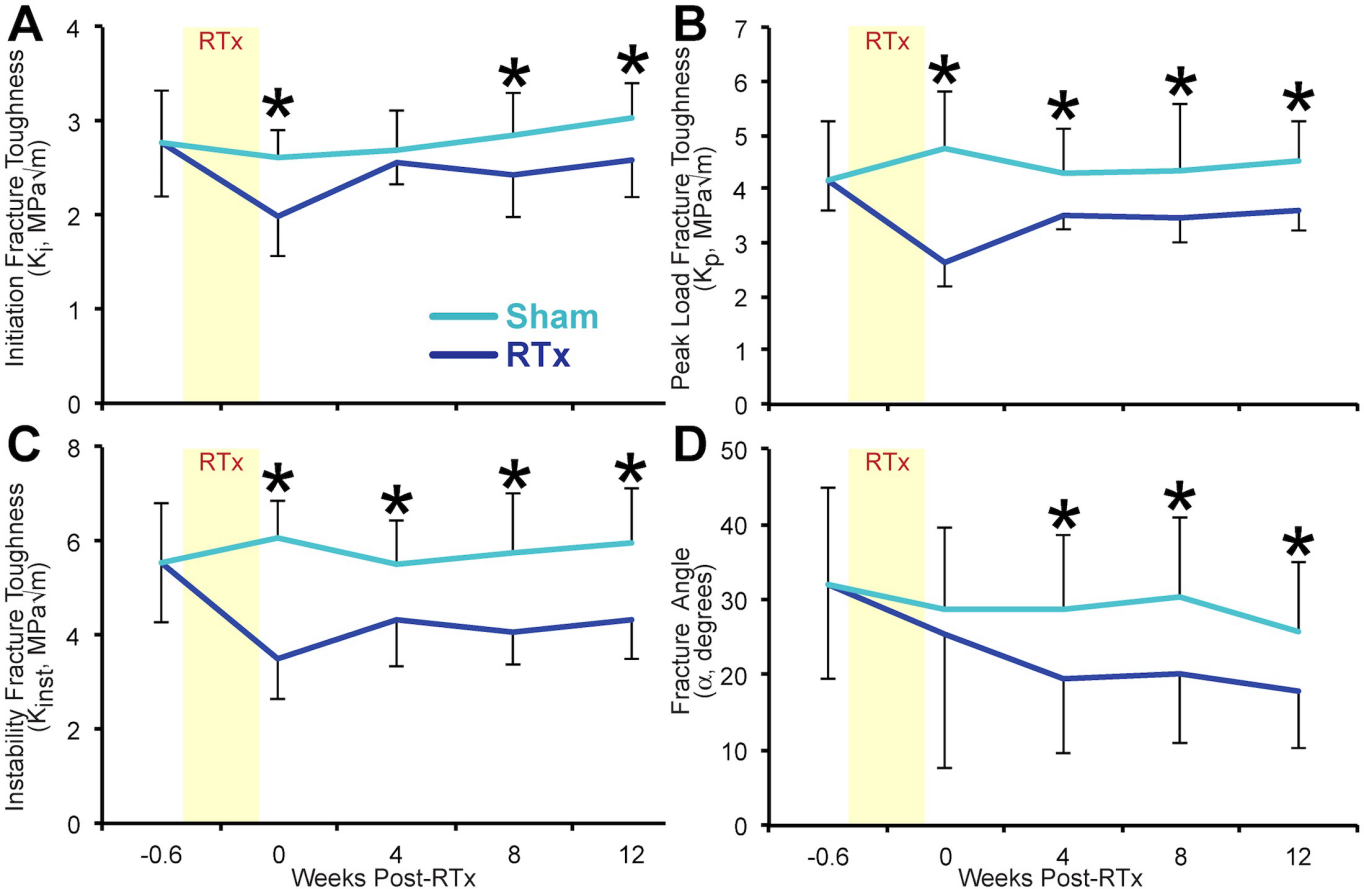
Fracture toughness outcomes. Fracture mechanics results for mid-diaphyseal cortical bone are presented as a function of time after treatment for RTX and Sham groups. (A) Initiation fracture toughness (K_init_); (B) peak load fracture toughness (K_pl_); (C) instability fracture toughness (K_inst_); and (D) fracture angle (α) are all decreased in RTx groups. Data are presented as mean ± standard deviation, n = 15 femurs/group/end point. Asterisks (*) denote p < 0.05 for Sham vs. RTx at each end point via an unpaired Student’s t-test.

Immediately following fractionated irradiation (week 0), the RTx group had decreased initiation fracture toughness (K_init_, -24%, p < 0.001), peak load fracture toughness (K_pl_, -45%, p < 0.001), and instability fracture toughness (K_inst,_ -42%, p < 0.001) relative to the Sham group (Fig 3A–3C). ANCOVA was used to test whether changes in fracture toughness over time differed between RTx and Sham femurs (Table 1). Overall, initiation toughness (K_init_) increased modestly with time (0.0377 MPa√m/wk, p < 0.001), but the slopes of the K versus time curves were not different for RTx and Sham groups (p = 0.731). There was a modest recovery in peak load fracture toughness (K_pl_) for the RTx group compared to Sham as indicated by the treatment*time interaction term (p = 0.021). However, even at 12 weeks, the K_pl_ was 20% lower in the RTx group (vs. Sham). The instability fracture toughness (K_inst_) did not change with time (p = 0.183) and the slopes of the RTx and Sham groups were not different (p = 0.178). Combined, these ANCOVA results show the initial post-RTx reduction in fracture toughness is not followed by further progressive loss of toughness. There is, however, no evidence of toughness recovery over the twelve week long study period, suggesting that the initial RTx-mediated reduction in fracture toughness is sustained long term.

**Table 1:** Analysis of covariance (ANCOVA) results.

Fracture toughness parameter	RTx	Time	RTx*Time	R^2^
Initiation (K_init_)	<0.001	<0.001	0.731	0.31
Peak (K_pl_)	<0.001	0.164	0.021	0.34
Instability (K_inst_)	<0.001	0.183	0.178	0.48
Fracture angle (α)	0.003	0.094	0.409	0.13

ANCOVA p-values for the effects of treatment (RTx) and time (weeks post-RTx) on the fracture mechanics parameters. RTx*Time is used to test for differences between slopes for the RTx vs. Sham groups. R^2^ indicates the overall model fit.

In addition to reduced crack growth resistance after RTx, the fracture pattern was more transverse in the RTx group (vs. Sham) as measured by a lower fracture angle. This reduction ranged from -30 to -34% for weeks 4–12 (p < 0.020, Fig 3D). These findings suggest that RTx may result in loss of toughening mechanisms that regulate crack initiation and growth in bone.

#### Cortical bone morphology

**Fig 4:**
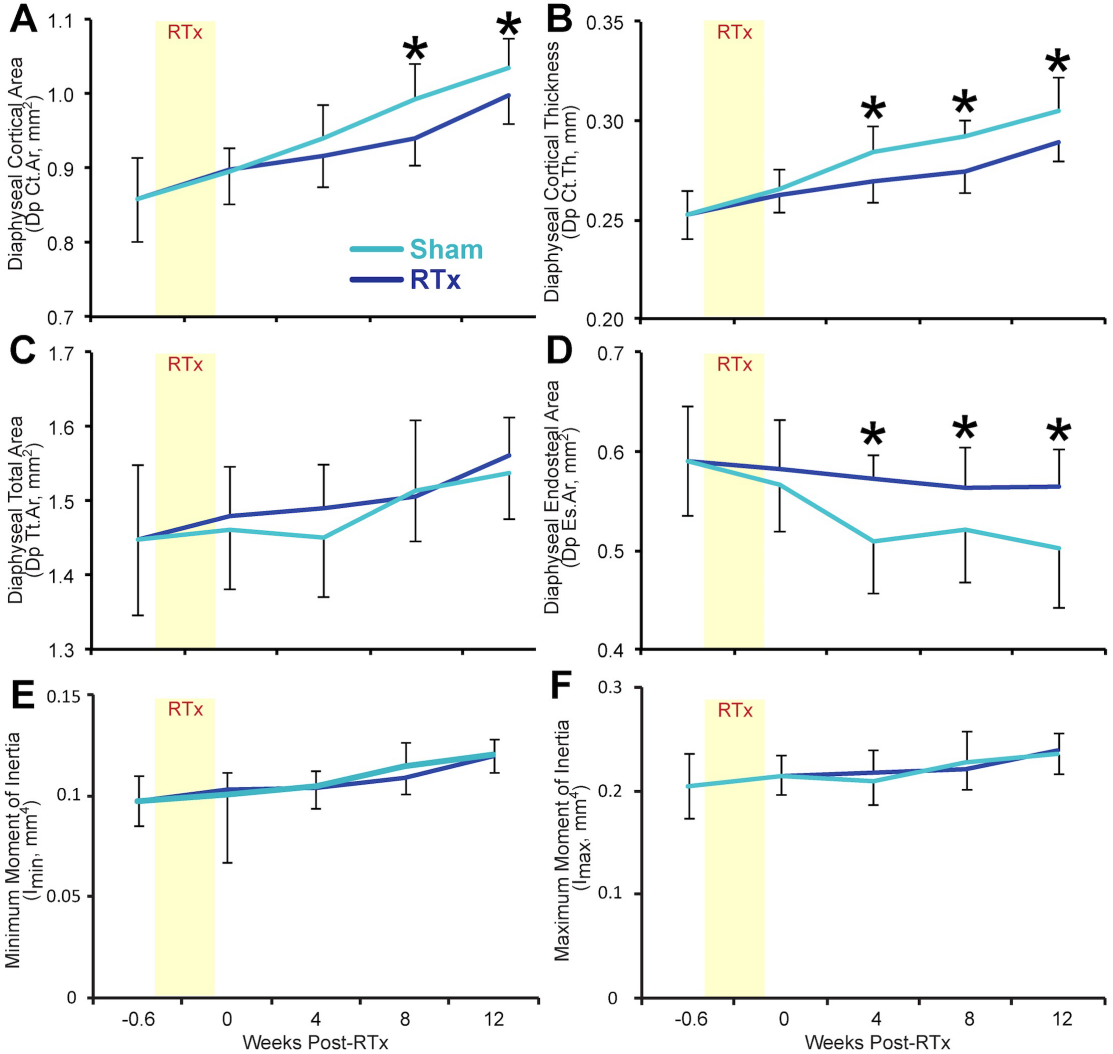
Mid-diaphyseal femur morphology. Mid-diaphyseal femur morphology is presented as a function of time after treatment for RTx and Sham groups. (A) Diaphyseal cortical cross-sectional area; (B) diaphyseal cortical thickness; (C) diaphyseal total cross-sectional area; (D) diaphyseal endosteal (marrow) cross-sectional area; (E) diaphyseal minimum moment of inertia (*I*_*min*_); and (F) maximum moment of inertia (*I*_*max*_). By 8–12 weeks RTx femurs lose cortical mass through resorption at the endosteal surface. Data are presented as mean ± standard deviation, n = 15 femurs/group/end point. Asterisks (*) denote p < 0.05 for Sham vs. RTx at each end point via an unpaired Student’s t-test.

In general, the relative changes in mid-diaphyseal cortical bone morphology following RTx were less dramatic compared to changes in bone fracture toughness. Cortical area reduced at 8 and 12 weeks for the RTx group (Dp Ct.Ar, -3.7 to -5.1%, p < 0.011) compared to the Sham group (Fig 4A). Cortical thickness was similarly decreased at 4, 8, and 12 weeks post-RTx (Dp Ct.Th, -5.1 to -6.2%, p < 0.007 vs. Sham) (Fig 4B). In contrast, irradiation did not significantly affect total area (Dp Tt.Ar) at any time point (Fig 4C). Endosteal area was increased at 4, 8, and 12 weeks post-RTx (Dp Es.Ar, +8.0 to 13%, p < 0.024) compared to Sham group (Fig 4D). The minimum moment of inertia (*I*_*min*_) and maximum moment of inertia (*I*_*max*_) (Fig 4F) were not different between the RTx and Sham groups.

#### Bone composition

**Fig 5:**
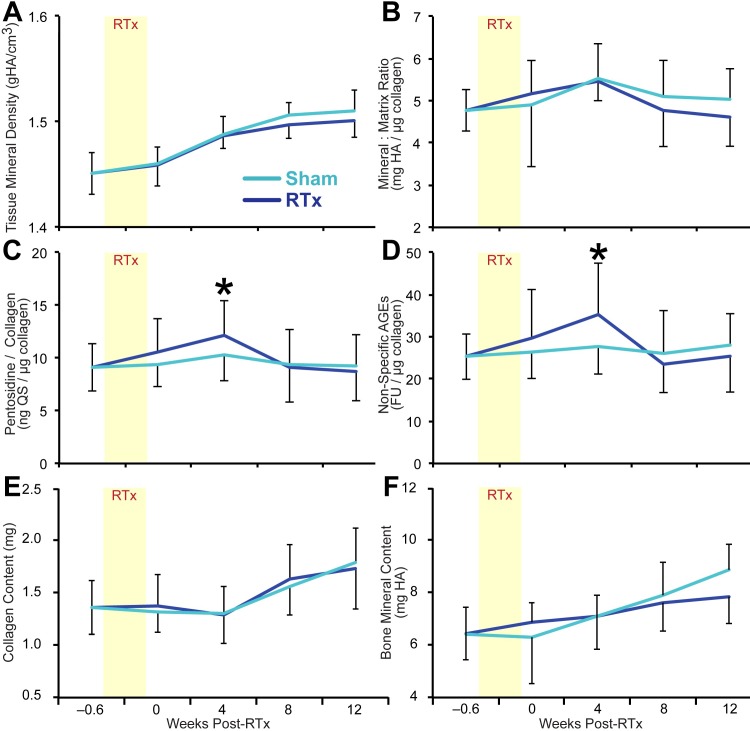
Bone biochemical composition. Biochemical composition of diaphyseal cortical bone is presented as a function of time after treatment for RTx and Sham groups. (A) Tissue mineral density; (B) mineral to matrix ratio; (C) pentosidine content normalized to collagen mass; (D) non-specific advanced glycation end products normalized to collagen mass; (E) collagen content; and (F) bone mineral content. Density, mineral:matrix ratio, collagen content, and bone mineral content did not differ between treatment groups, and AGE content for RTx femurs is increased only at 4 weeks in this data set. Data are presented as mean ± standard deviation, n = 15 femurs/group/end point. Asterisks (*) denote p < 0.05 for Sham vs. RTx at each end point via an unpaired Student’s t-test.

In contrast to the fracture toughness outcome measures, bone composition parameters were not strongly affected by RTx. Tissue mineral density (TMD), mineral:matrix ratio, collagen content, and bone mineral content did not differ between RTx and Sham groups (Fig 5A and 5B). Pentosidine (+21%, p = 0.030) and nsAGE (+30%, p = 0.049) content (normalized to collagen) were increased only at 4 weeks post-RTx (vs. Sham) (Fig 5C and 5D). There were no significant correlations between bone composition measures (pentosidine, nsAGEs) and any of the fracture toughness outcomes (e.g., for K_init_ versus nsAGEs: R^2^ = 0.025, p = 0.15).

### Hypothesis 2: RTx damages bone directly

#### Evaluation of devitalized bone fracture toughness

**Fig 6:**
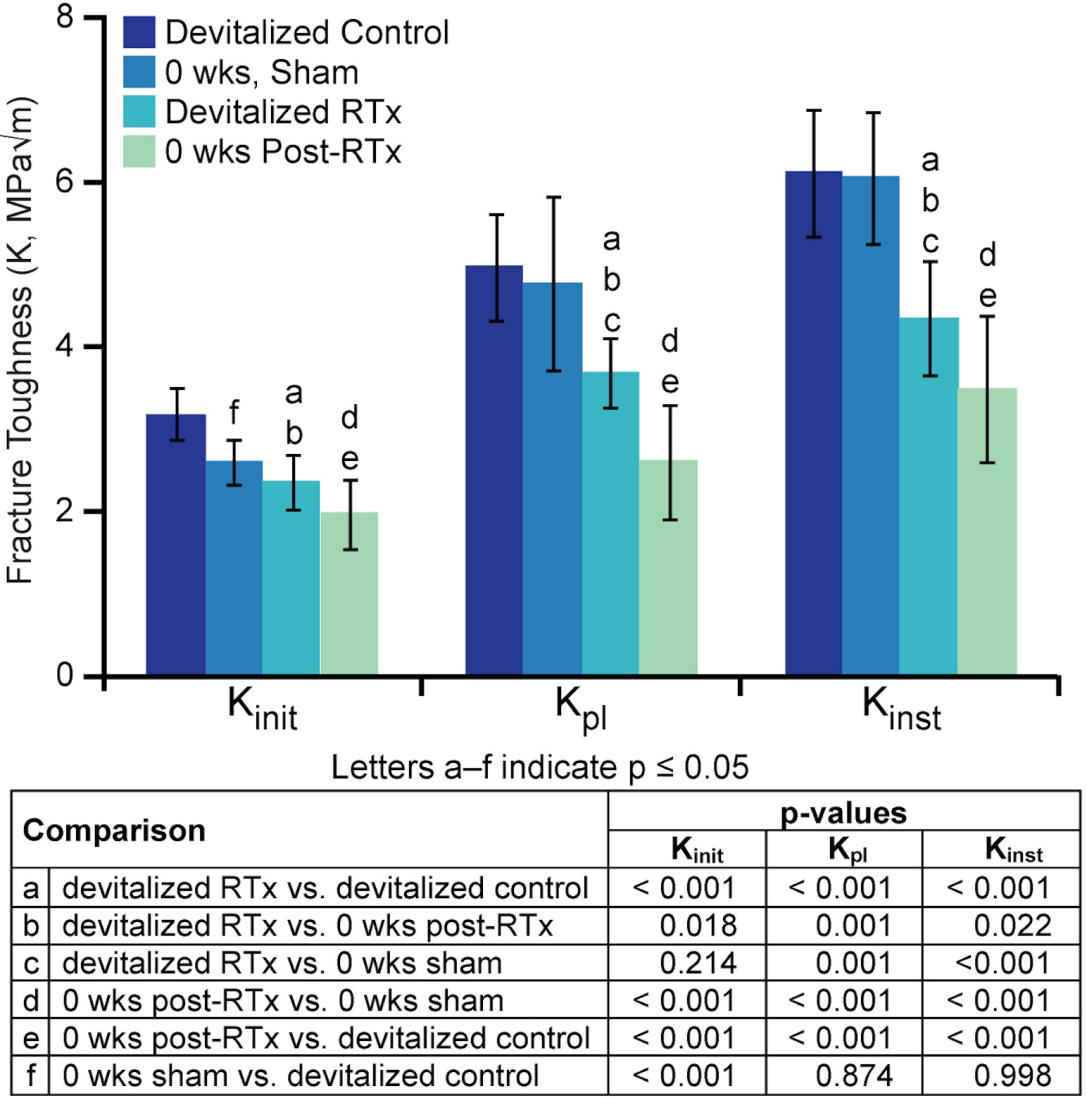
Fracture toughness of bone irradiated *ex vivo*. Mouse cadaver femurs were devitalized and exposed to 0 Gy or 20 Gy X-irradiation. Fracture mechanics outcomes—including initiation (K_init_), peak load (K_pl_), and instability (K_inst_) toughness measures—are presented alongside data from the *in vivo* groups (Sham, RTx) at 0 weeks. Data is presented as mean ± standard deviation, n = 15 femurs/group. Lower case letters denote statistically significant comparisons (p < 0.05) between treatment groups via one-way ANOVA with Tukey’s post-hoc test. Exact p-values are listed in the accompanying table.

Irradiation of devitalized mouse femurs resulted in significantly decreased K_init_ (-26%, p < 0.001), K_pl_ (-26%, p < 0.001), and K_inst_ (-29%, p < 0.001) compared to non-irradiated contralateral control femurs (Fig 6). Crack deflection angle was not significantly different (p = 1.00) between irradiated (11.3° ± 4.5°) and contralateral (11.4° ± 4.0°) femurs. When comparing devitalized RTx femurs to 0 week post-RTx *in vivo* femurs, the latter had lower K_init_ (-16%, p = 0.018), K_pl_ (-29%, p = 0.001), and K_inst_ (-18%, p = 0.022), suggesting the magnitude of the changes *in vivo* were greater than those in the devitalized bone (Fig 6). Interestingly, at 0 weeks the Sham femurs had a lower initiation toughness (-18%, p < 0.001) than devitalized controls, with no difference in K_pl_ or K_inst_ (Fig 6). The reduction in fracture toughness in these *ex vivo* irradiated devitalized specimens indicate that radiation therapy directly decreases bone fracture toughness.

### Contribution of AGEs to decreased toughness post-RTx

#### Evaluation of artificially ribosylated bone

**Fig 7:**
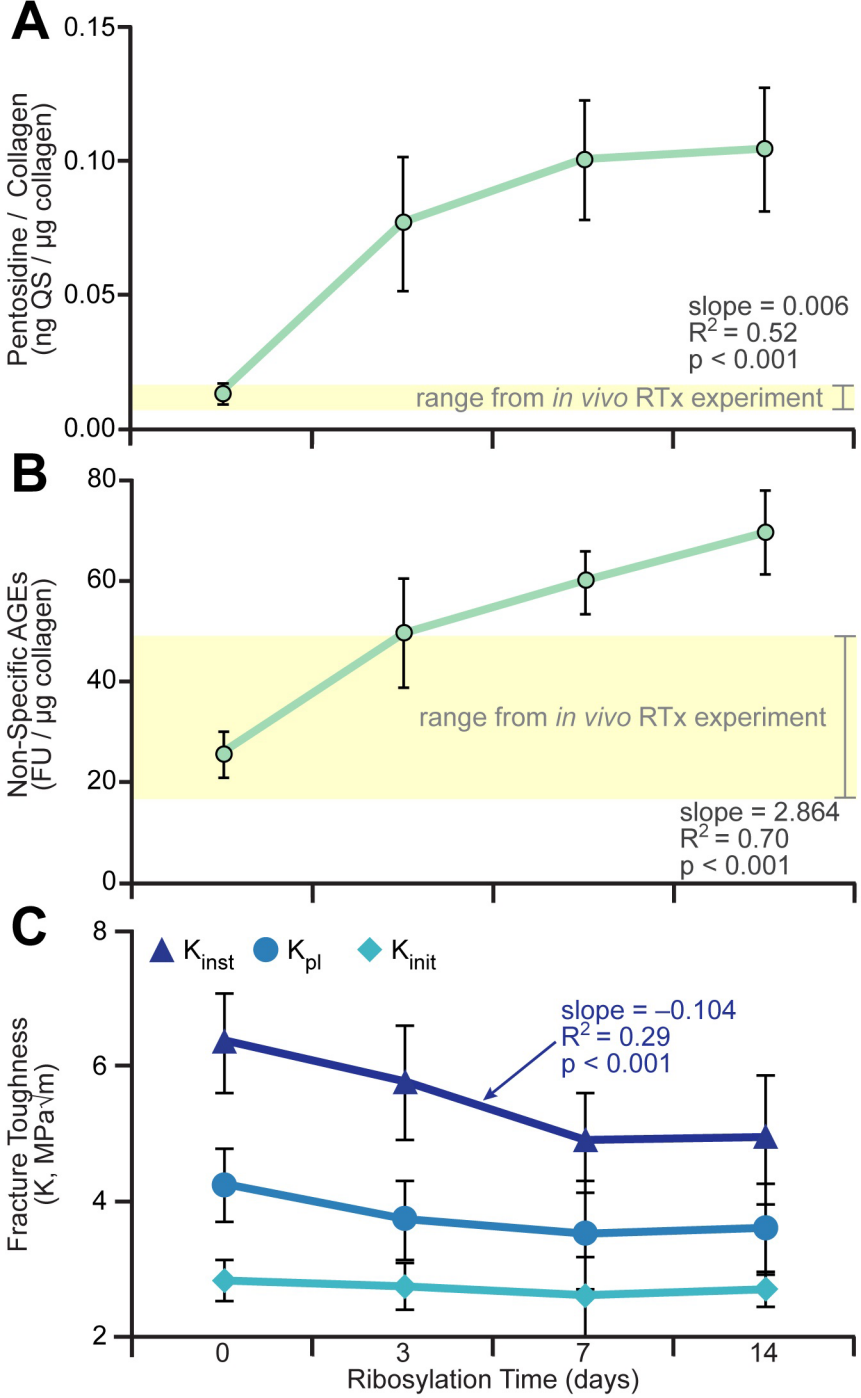
*Ex vivo* glycation of bone and associated changes in fracture toughness. To explore the role of AGEs in fracture toughness, mouse femurs were incubated in a ribose solution for 0–14 days *ex vivo* to induce formation of AGEs. (A) Pentosidine content normalized to collagen mass; (B) non-specific AGEs content normalized to collagen mass; (C) fracture toughness outcomes. K_inst_ was negatively correlated with ribosylation, but at 3–14 days in ribose solution, the femur AGE content supraphysiologic. Data are presented as mean ± standard deviation, n = 15 femurs/group/end point. Simple linear regression was used to determine correlations between toughness or composition and ribosylation time.

Predictably, pentosidine content increased with longer incubation time in the ribosylation solution (slope = 0.006 μg QS/μg collagen/day of ribosylation, R^2^ = 0.52, p < 0.001), as did nsAGEs (slope = 2.864 FU/μg collagen/day of ribosylation, R^2^ = 0.70, p < 0.001, n = 9–10 samples/group) (Fig 7A and 7B). Also as expected, instability fracture toughness decreased with increased incubation time in ribose solution (slope = -0.104 MPa√m)/day of ribosylation, R^2^ = 0.29, p < 0.001) (Fig 7C). Instability fracture toughness was negatively correlated with nsAGE (slope = -0.033 MPa√m/FU/μg collagen, R^2^ = 0.334, p < 0.001) and pentosidine contents (slope = -14.016 MPa√m/μg QS/μg collagen, R^2^ = 0.334, p < 0.001). However, initiation toughness (slope = -0.010 MPa√m)/day of ribosylation, R^2^ = 0.017, p = 0.42) and peak load toughness (slope = -0.040 MPa√m)/day of ribosylation, R^2^ = 0.089, p = 0.064) did not decrease as a function of days of ribosylation.

## Discussion

This work provides direct and definitive evidence that radiotherapy results in diminished bone fracture toughness. Data presented here suggest that the decreased ability to resist crack initiation and growth post-RTx plays a greater role in fracture risk than the accompanying changes in bone morphology and composition (S2 Document). In this hindlimb irradiation model, cortical bone toughness was lost very early after RTx, but no further longitudinal, progressive toughness reduction occurred (as we had initially proposed in our first hypothesis). Potential factors contributing mechanistically to post-radiation loss of fracture toughness include cell-mediated changes in bone morphology or composition, and direct (non-cell-mediated) radiation damage to bone. Each of these potential mechanisms was evaluated in this paper. The *in vivo* portion of the experiment evaluated the overall effects of radiation on cortical bone fracture toughness and geometry over time. These changes were compared to the direct effects of RTx on fracture toughness in the devitalized bone experiment. The latter demonstrated a measurable direct effect of radiation on fracture toughness but to a lesser degree than *in vivo*. Finally, the artificial ribosylation study showed that the magnitude of matrix glycation required to alter fracture mechanics—as measured here—greatly exceeds the amount of glycation induced by our hindlimb irradiation protocol.

There were several limitations in the animal model and mechanical testing approach used in this study. Using the bilateral hindlimb RTx model results in exposure of a much larger percentage of the mouse than is used clinically in human radiotherapy, possibly soliciting a greater systemic response to radiation than would be clinically expected. The mechanical test used a single loading cycle instead of cyclic fatigue loading, which would accumulate damage in the bone prior to fracture [41]. The calculations of fracture toughness here assume that the 3D crack front is flat and symmetrical through the crack growth process. The crack front was only documented at one location (medial surface). Further, the closed form solution used here assumes the bone is a cylinder, while the actual cross section is elliptical, and not completely symmetrical [24, 25]. Finally, while the reflected white light method for measurement of crack front propagation used here enabled us to evaluate a large number of femurs in a time-efficient manner, the resulting fracture toughness measurements may not be as precise as those measured with environmental scanning electron microscopy.

In this study, we found it possible to document progression of crack growth during loading using very high resolution reflected white light imaging. A similar approach was also recently used to document crack extension in rat tibia [35]. While reflected white light imaging does not have the resolution of SEM, it may be advantageous for cases where a very large number of test specimens (n = 135 in this study) are needed for statistical power considerations. The fracture toughness values and coefficients of variation (COV) determined here for the BALB/cJ Sham-treated mice (K_inst_ = 5.5 MPa√m, COV = 0.14) were similar to that determined for C57BL/6J mice (K_inst_ = 4.6 MPa√m, COV = 0.13) using SEM imaging [24]. It should be noted that the C57BL/6J mice have lower bone mineral density compared to the BALB/cJ mice. Overall, these findings suggest that it is possible to obtain consistent results using the white light imaging method for this mouse model.

The linear elastic fracture mechanics (LEFM) approach used here assumes that the plastic zone around the crack tip is small in comparison to the local geometry and crack length. Estimates of plastic zone size (assuming yield strength of 160 MPa [11]) were found to be valid for the smaller K values (< 3 MPa√m) with this BALB/cJ mouse model. For higher K values including the peak load and instability fracture toughness values presented here, the LEFM assumption is not strictly valid. The same conclusion was found for C57BL/6J mice [25]. However, in both these instances, relative comparisons between treatment groups should still be valid.

**Fig 8:**
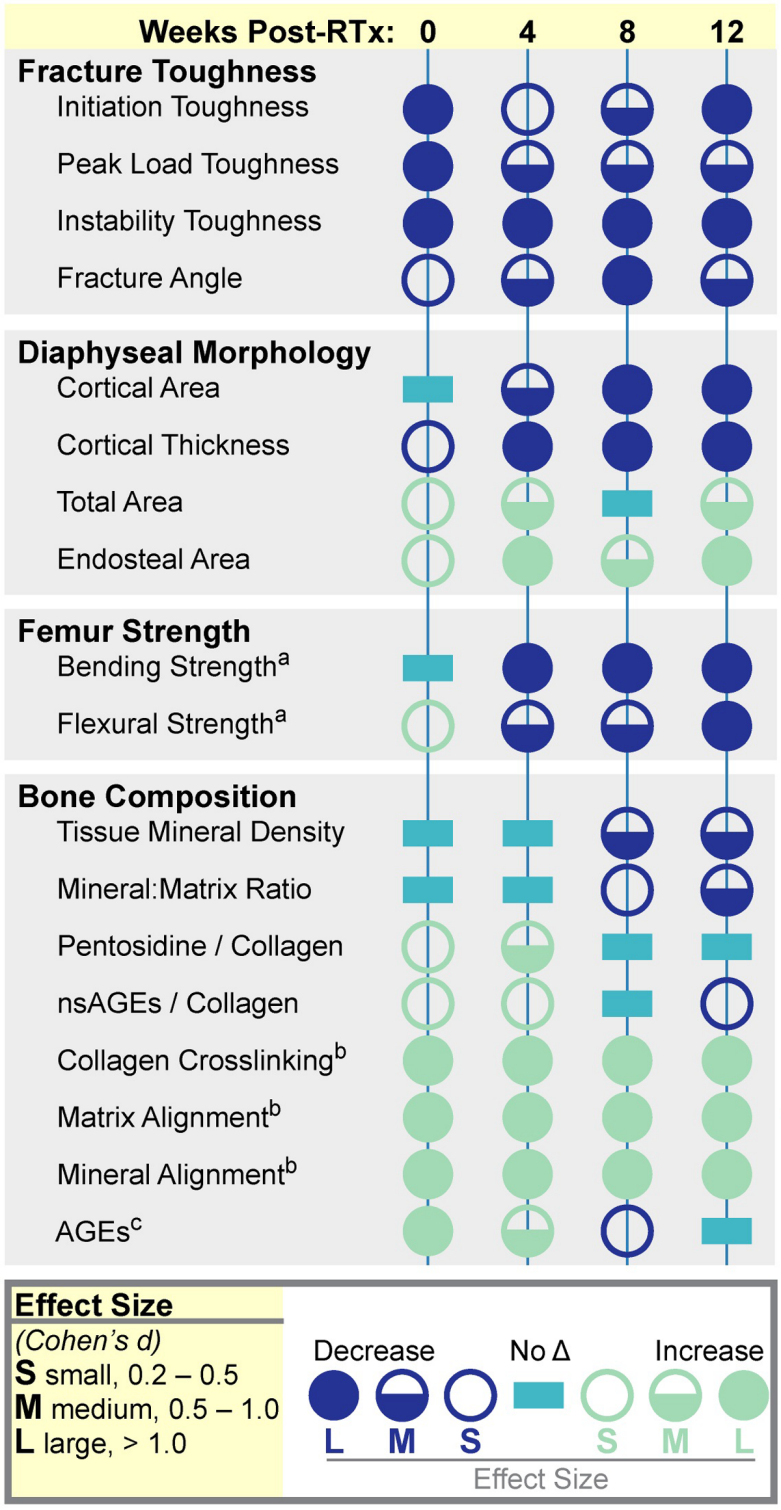
Known effects of limited field irradiation on murine bone. In this summary figure, longitudinal changes in bone fracture toughness, morphology, whole bone mechanics, and tissue composition are presented as effect size (Cohen’s d method). Data are derived from the study presented in this manuscript, as well as historical data: ^a^Oest ME, JBMR 2017 [11]; ^b^Oest ME, Bone 2016 [27]; ^c^Oest ME, Radiat Res 2014 [26].

To provide an overall picture of the changes in biomechanics, morphology, and biochemistry results post-RTx from this study as well as our lab’s previous studies, Fig 8 was created using statistical effect size (mean difference/standard deviation) as a generic outcome measure. Cohen’s d formula values were grouped into small, medium, and large effect size categories. The finding of decreased material fracture toughness builds upon our previous study, which showed through structural (finite element) analysis that post-RTx loss of bone quantity and structure do not fully explain bone fragility [14]. Using a strength of materials approach, we have also previously demonstrated decreased structural bending strength and bone material (flexural) strength post-RTx (Fig 8) [11]. These data suggest that radiation can reduce strength by diminishing bone quality independent of morphology and tissue mineral density. Potential contributors to these alterations in bone quality include the mineral and organic matrix components and their biochemistry.

Both mineral and organic matrix components influence bone fracture resistance. While the mineral phase primarily influences fracture initiation [42, 43], organic matrix quality impacts fracture propagation [28, 29, 42, 44, 45]. In this study, post-RTx reduction of fracture initiation toughness does not appear to be explained by changes in tissue mineral density (TMD), consistent with clinical observations that bone mineral density does not change following RTx [46, 47]. This could implicate a role for post-irradiation damage to the mineral-collagen interface, further supported by the fact that bone mineral density (TMD) was not altered after radiation. Previous studies using Raman have demonstrated early and sustained hyperalignment of collagen and mineral crystals following RTx (Fig 8) [27]. In addition to tissue composition and organization, modifications to matrix biochemistry may contribute to post-radiotherapy bone fragility. Raman spectroscopy analysis has also shown early and sustained changes in divalent:trivalent enzymatic collagen crosslink ratio (Fig 8) [27, 48], suggesting collagen chemistry may be implicated in post-radiotherapy loss of fracture toughness.

AGEs are another collagen modification potentially affecting toughness following RTx. Increased enzymatic crosslinking and AGE formation have been associated with reduced bone fracture toughness in models of diabetes and aging [30, 38, 49–55]. Because AGEs accumulation can be induced by oxidative stress [56–58] following generation of primary and secondary reactive oxygen species via ionizing radiation [59–62], it seemed logical that AGEs might contribute to post-irradiation bone embrittlement. However, in this study a small (but significant) increase in pentosidine and nsAGEs was seen only at four weeks post-irradiation, while a previous study showed increased pentosidine and nsAGEs one week post-RTx (Fig 8) [26]. While AGE content likely affects the material properties of the bone at some level, AGE content alone cannot account for the RTx-induced loss of fracture toughness seen here. These data suggest that other tissue compositional changes are involved. The specific contributions of AGEs and other matrix changes to post-radiotherapy bone embrittlement merit further investigation. The early reduction in fracture toughness shown here suggests alterations to bone tissue constituents (mineral and/or collagen) can occur immediately after RTx.

The radiation-induced decrease in fracture toughness (K_init_, K_pl_) demonstrated in the devitalized bone experiment provides highlights the role of direct, non-cell-mediated radiation damage to increased bone fragility following radiotherapy. While it is known from bone allograft sterilization studies that high doses of radiation (kGy range) weaken bone [63–72], this result has not previously been demonstrated in the therapeutic radiation dose range. We are, however, pursuing the possibility of collagen fragmentation following therapeutic RTx doses in future studies, as this could potentially contribute to bone matrix embrittlement. In addition to collagen fragmentation (bond cleavage), radiation may directly damage bone tissue through generation of reactive oxygen species [59] that subsequently oxidize proteins, fats, and sugars within the tissue [73]. Interestingly, while radiation reduced toughness in both the devitalized and *in vivo* samples, loss of toughness for *in vivo* samples exceeded that of the devitalized irradiated tissues (Fig 6). These data indicate an additive effect, wherein direct radiation damage is accompanied by cell-mediated processes, with both mechanisms contributing to post-radiotherapy bone fragility. This amplification of RTx-induced bone damage *in vivo* could be effected via oxidative stress and inflammatory responses, including generation of secondary reactive oxygen species [61, 74]. The fracture angle data further highlights a role for ongoing cellular regulation of bone fragility; no change in fracture angle is noted until the week four end point. This suggests that, over time, local cell-mediated tissue remodeling alters material toughening mechanisms, resulting in more linear crack propagation. Previous work in this model has demonstrated continued appositional bone formation post-RTx [31], as well as longitudinal changes in mineral:matrix ratio, collagen crosslink maturity, and mineral crystallinity [27, 48]. While the *in vivo* experiment demonstrated a greater degree of radiation-associated loss of bone toughness, the *in vitro* data indicate that direct RTx-mediated bone damage—in the absence of cellular activity—meaningfully contributes to loss of bone strength.

To directly explore the extent of glycation needed to directly affect fracture toughness, mouse femurs were artificially ribosylated to induce AGE accumulation within the tissue. As expected, incubation of femurs in a ribose solution increased the concentration of pentosidine and nsAGEs in a time-dependent manner until a saturation point was reached. The reduced instability fracture toughness of the femurs demonstrates that glycation contribute to bone embrittlement. However unlike RTx, ribosylation did not reduce the toughness (K_init_, K_pl_) or crack deflection angle, further indicating that AGE accumulation may not be the primary embrittlement mechanism in irradiated bone. Other studies support this data by similarly showing ribosylation-induced reductions in fracture propagation toughness but no effects on initiation toughness [42, 75]. The AGE content necessary to decrease instability toughness (7–14 days incubation) greatly exceeded the maximal AGE content of bones irradiated *in vivo*, and is likely supraphysiologic (highlighted by the horizontal yellow boxes in Fig 7B and 7C).

The clinical relevance of this *in vivo* model his highlighted by two results. First, the failure of fracture toughness to recover by twelve weeks post-RTx—coupled with decreased bone quantity at late time points—is consistent with the late-onset nature of clinical radiotherapy-associated fragility fractures. These fractures typically occur months to years following the completion of radiotherapy. Second, the reduced fracture angle in irradiated samples is consistent with clinical observations of radiotherapy-associated fragility fractures occurring most frequently as transverse fractures [76]. Since crack deflection is an important toughening mechanism in lamellar bone, reduced crack deflection may facilitate fracture by permitting more rapid crack growth with less energy required for crack propagation [77, 78]. Other bone toughening mechanisms at smaller length scales may also be affected by radiation, but not studied herein, include microcracking, ligament bridging, collagen fibril bridging, and fibrillar sliding.

Overall this study shows that bone quality—specifically fracture toughness—is reduced early following radiation in both living and devitalized models. It also demonstrates failure of bone quality to recover *in vivo*. The greater decrease in fracture toughness post-RTx observed *in vivo* (vs. devitalized specimens) highlights a role for additive contributions of both direct and cell-mediated bone matrix damage mechanisms. There were no significant correlations between the fracture toughness and bone composition outcomes measured here (AGEs, TMD, or mineral:matrix ratio). However, previous measures of enzymatic collagen crosslinking, matrix alignment, and mineral alignment all had sustained increases, which may be related to the decreased toughness found here. The exact mechanism leading to the reduction of fracture propagation resistance merits further investigation in order to provide a basis for better prevention and treatment.

## Supporting information

S1 Table Final body weight of mice after radiation. (DOCX)

S1 Document Approach used to calculate stress intensity factor (K) using a closed form solution of a crack in a thick-walled cylinder loaded in three point bending. (DOCX)

S2 Document Parametric analyses of bone morphology and toughness on femur strength. (DOCX)

S3 Document ARRIVE guidelines checklist. (PDF)
