# Supplementary material for: Limited field radiation therapy results in decreased bone fracture toughness in a murine model
Source: PLoS One. 2018 Oct 3;13(10):e0204928. doi: 10.1371/journal.pone.0204928 (PMC6169919; doi:10.1371/journal.pone.0204928)
Supplement: S1 Table — (DOCX) [file pone.0204928.s001.docx]

**S1 Table. Final body weight of mice after radiation**

|  | **Final Body Mass (g)** | | | | |
| --- | --- | --- | --- | --- | --- |
| **End Point** | **–4 days** | **0 weeks** | **4 weeks** | **8 weeks** | **12 weeks** |
| **Sham** | 20.8 ±1.8 | 21.0 ±1.6 | 22.1 ±1.1 | 24.7 ±2.3 | 26.0 ±1.7 |
| **RTx** |  | 21.4 ±1.1 | *23.3 ±1.4 | *22.9 ±1.3 | 26.3 ±1.8 |

Mean (± standard deviation) body mass of mice at each end point of the study. Asterisks (*) indicate p < 0.05 vs. Sham group within a time point via an unpaired Student’s t-test.
