# Supplementary material for: Limited field radiation therapy results in decreased bone fracture toughness in a murine model
Source: PLoS One. 2018 Oct 3;13(10):e0204928. doi: 10.1371/journal.pone.0204928 (PMC6169919; doi:10.1371/journal.pone.0204928)
Supplement: S1 Document — (DOCX) [file pone.0204928.s002.docx]

**Supplemental Document 1**

**Approach used to calculate stress intensity factor (K) using a closed form solution of a crack in a thick-walled cylinder loaded in three point bending**

The approach used here follows that described by Carriero et al. The projected crack length (a_proj_) was determined from the high-resolution images on the medial side of the diaphysis (**Fig S1A**) during the loading of the femur. The geometry of the cross section was determined post-fracture using images of the transverse sections of the diaphysis. Following length calibration, the periosteal width (W_p_), periosteal height (H_p_), endosteal width (W_e_), and endosteal height (H_e_) were measured. From these, the geometry of an average thick cylinder was calculated with outer (R_o_), inner (R_i_), and mean (R_m_) radii:

R_o_ = (H_p_ + W_p_) / 2

R_i_ = (H_e_ + W_e_) / 2

R_m_ = (R_o_ + R_i_) / 2

To account for scaling of cross section with H_p_ < 2R_o_, a modified projected crack length (**Fig S1B**) is calculated (a’):

a’ = 2 R_o_ a_proj_ / H_p_

From trigonometric considerations, the instantaneous half crack angle (θ_inst_) can be calculated:

θ_inst_ = cos^-1^ ((R_o_ – a’) / R_m_)

As described by Carriero et al, these parameters are used to calculated F_b_, which is a geometry parameter for the stress intensity calculation accounting for geometry and crack length.

where:

A_b_ = 0.65133 – 0.5774*e* – 0.3427*e*^2^ – 0.0681*e*^3^

B_b_ = 1.879 + 4.975*e* + 2.343*e*^2^ – 0.6197*e*^3^

C_b_ = –9.779 – 38.14*e* – 6.611*e*^2^ + 3.972*e*^3^

D_b_ = 34.56 + 129.9*e* + 50.55*e*^2^ + 3.374*e*^3^

E_b_ = –30.82 – 147.6*e* – 78.38*e*^2^ – 15.54*e*^3^

*e* = log (t/R_m_)

and t is the thickness of the cylinder: (R_o_ – R_i_).

Finally, the LEFM stress-intensity (K) can be calculated:

Where P is the applied load and S is the span. All other parameters are described above. As noted by Carriero et al, the solution is valid for a maximum instantaneous half crack angle of 110°.


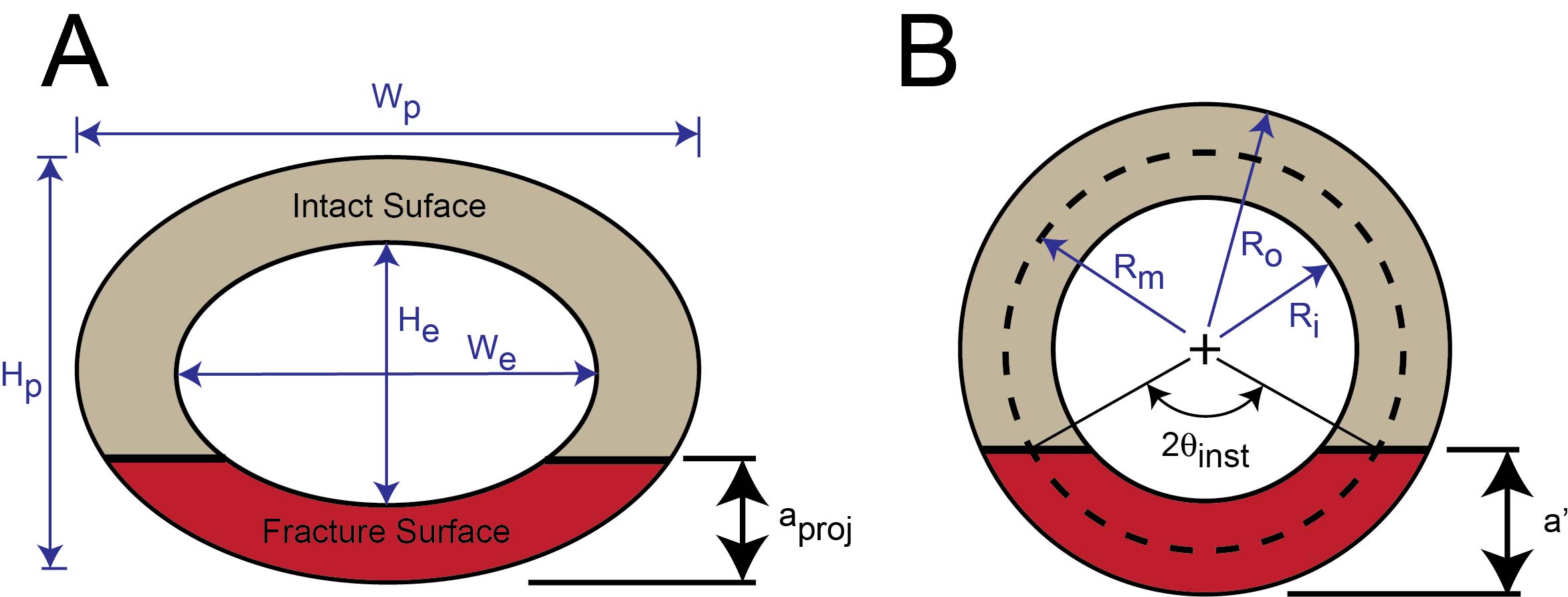


**Figure S1. Geometric considerations to calculate instantaneous half crack angle.** (A) The cross sectional geometry at the diaphysis was measured to document periosteal height (H_p_) and width (W_p_), and endosteal height (H_e_) and width (W_e_). The projected height of the fracture surface (a_proj_) was documented during loading. (B) An idealized cylindrical geometry was used with outer (R_o_), inner (R_i_), and mean (R_m_) radii calculated from the sectional height/width measures. A modified projected crack length (a’) was calculated as a proportion of the a_proj_ to H_p_ measure.
