# Supplementary material for: Limited field radiation therapy results in decreased bone fracture toughness in a murine model
Source: PLoS One. 2018 Oct 3;13(10):e0204928. doi: 10.1371/journal.pone.0204928 (PMC6169919; doi:10.1371/journal.pone.0204928)
Supplement: S2 Document — (DOCX) [file pone.0204928.s003.docx]

**Supplemental Document 2**

**Relative effect of changes in morphology and fracture toughness on femur bending strength: A parametric study**

**Introduction**

Both bone size and material fracture toughness of the diaphyseal cortical bone can be affected by RTx treatment. To provide an estimate of the relative effects of these changes on femur bending strength, a numerical parametric study was performed using a hollow beam with a notch under three point bending conditions.

**Methods**

The force required to initiate crack propagation to a femur with a notch (25% of height) loaded in three-point bending was calculated using the closed form solution for fracture toughness of a notched cylinder [1, 2]. Three cases were considered. First, the reduction in load needed to initiate a crack for changes in diaphyseal geometry alone were determined for the RTx group versus sham group. Second, the load required to initiate crack growth for the case where there was only a change in bone material fracture toughness was considered. Finally, including both morphology and material fracture toughness changes was explored. Mean cortical thickness (Ct.Th), radius (Ct.Rm) and initiation toughness (K_i_) for each time point, and treatment were used as input variables for the analysis.

**Results**

There was a modest reduction in load needed to initiate a crack due to changes in diaphyseal geometry alone following RTx (up to 5%, S2 Table). There was a larger reduction in load required to initiate a crack growth (up to 31%) due to reduced bone material toughness for the RTx group. Finally, including both morphology and material changes shows an additive effect in the loss of load needed to initiate a crack. Overall, these results show that in this mouse model with RTx, the effect of loss of bone strength due to reduction in material toughness is greater than that due to loss in bone size.

**Table S2. Parametric analyses of bone morphology and toughness. Geometry (Ct.Rm, Ct.Th) and initiation toughness (Ki) are input parameters based on experiments**

| **Weeks** | | | **0** | **4** | **8** | **12** |
| --- | --- | --- | --- | --- | --- | --- |
| Δ in Mid-Diaphyseal Morphology | Ct.R_m_ (mm) | Sham | 0.553 | 0.541 | 0.551 | 0.549 |
|  |  | RTx | 0.558 | 0.558 | 0.558 | 0.564 |
|  | Ct.Th (mm) | Sham | 0.257 | 0.277 | 0.286 | 0.300 |
|  |  | RTx | 0.255 | 0.260 | 0.268 | 0.281 |
|  | **Predicted Δ in Load (%)** | | **0.5** | **–2.9** | **–5.2** | **–3.1** |
| Δ in Fracture Toughness | K_i_ (MPa√m) | Sham | 2.61 | 2.69 | 2.85 | 3.02 |
|  |  | RTx | 1.99 | 2.57 | 2.37 | 2.59 |
|  | **Predicted Δ in Load (%)** | | **–31.2** | **–4.7** | **–20.3** | **–16.6** |
| Δ in Combined Morphology & Toughness | **Predicted Δ in Load (%)** | | **–30.5** | **–7.8** | **–26.9** | **–20.3** |

Parametric analyses of the effects of changing bone morphology and fracture toughness on the load required to initiate crack propagation for an idealized cylindrical bone with a defect were performed. The relative change in load required to initiate fracture (Δ Load (%) = [RTx – Sham]/Sham) was determined for cases where there is only a change in morphology, only a change in bone fracture toughness, and combined changes in morphology and toughness. Abbreviations: Ct.R_m_, mean cortical radius; Ct.Th, mean cortical thickness; K_i_, initiation toughness.

**Discussion**

In addition to bone material properties and biochemistry, morphology changes after radiation can have an effect on femur strength. Because both the morphology and material toughness change following RTx, we explored the relative effects of these changes on bone strength for a cylindrical bone with a notch under 3-point bend. On average the combined effect of diaphyseal morphology and material toughness reduced the predicted fracture initiation load by 21%. Diaphyseal geometric changes alone were responsible for a 2.7% reduction in predicted fracture initiation load, and material toughness changes alone were responsible for an 18% reduction in predicted fracture initiation load. Loss of material toughness had a much larger effect on bone strength than changes in diaphyseal geometry.

**References**

1. Ritchie RO, Koester KJ, Ionova S, Yao W, Lane NE, Ager JW, 3rd. Measurement of the toughness of bone: a tutorial with special reference to small animal studies. Bone. 2008;43(5):798-812. doi: 10.1016/j.bone.2008.04.027. PubMed PMID: 18647665; PubMed Central PMCID: PMCPMC3901162.

2. Carriero A, Zimmermann EA, Shefelbine SJ, Ritchie RO. A methodology for the investigation of toughness and crack propagation in mouse bone. J Mech Behav Biomed Mater. 2014;39:38-47. doi: 10.1016/j.jmbbm.2014.06.017. PubMed PMID: 25084121.

3. Oest ME, Policastro CG, Mann KA, Zimmerman ND, Damron TA. Longitudinal Effects of Single Hindlimb Radiation Therapy on Bone Strength and Morphology at Local and Contralateral Sites. J Bone Miner Res. 2017. doi: 10.1002/jbmr.3289. PubMed PMID: 28902435.

4. Oest ME, Franken V, Kuchera T, Strauss J, Damron TA. Long-term loss of osteoclasts and unopposed cortical mineral apposition following limited field irradiation. J Orthop Res. 2015;33(3):334-42. doi: 10.1002/jor.22761. PubMed PMID: 25408493; PubMed Central PMCID: PMCPMC4382807.

5. Willey JS, Lloyd SA, Robbins ME, Bourland JD, Smith-Sielicki H, Bowman LC, et al. Early increase in osteoclast number in mice after whole-body irradiation with 2 Gy X rays. Radiat Res. 2008;170(3):388-92. doi: 10.1667/RR1388.1. PubMed PMID: 18763868; PubMed Central PMCID: PMCPMC2597156.
